# Enhancing simulations with intra-subject variability for improved psychophysical assessments

**DOI:** 10.1371/journal.pone.0209839

**Published:** 2018-12-31

**Authors:** Mike D. Rinderknecht, Olivier Lambercy, Roger Gassert

**Affiliations:** Rehabilitation Engineering Laboratory, Institute of Robotics and Intelligent Systems, Department of Health Sciences and Technology, ETH Zurich, Zurich, Switzerland; The University of Melbourne, AUSTRALIA

## Abstract

Psychometric properties of perceptual assessments, like reliability, depend on stochastic properties of psychophysical sampling procedures resulting in method variability, as well as inter- and intra-subject variability. Method variability is commonly minimized by optimizing sampling procedures through computer simulations. Inter-subject variability is inherent to the population of interest and cannot be influenced. Intra-subject variability introduced by confounds (e.g., inattention or lack of motivation) cannot be simply quantified from experimental data, as these data also include method variability. Therefore, this aspect is generally neglected when developing assessments. Yet, comparing method variability and intra-subject variability could give insights on whether effort should be invested in optimizing the sampling procedure, or in addressing potential confounds instead. We propose a new approach to estimate intra-subject variability of psychometric functions by combining computer simulations and behavioral data, and to account for it when simulating experiments. The approach was illustrated in a real-world scenario of proprioceptive difference threshold assessments. The behavioral study revealed a test-retest reliability of *r* = 0.212. Computer simulations without considering intra-subject variability predicted a reliability of *r* = 0.768, whereas the new approach including an intra-subject variability model lead to a realistic estimate of reliability (*r* = 0.207). Such a model also allows computing the theoretically maximally attainable reliability (*r* = 0.552) assuming an ideal sampling procedure. Comparing the reliability estimates when exclusively accounting for method variability versus intra-subject variability reveals that intra-subject variability should be reduced by addressing confounds and that only optimizing the sampling procedure may be insufficient to achieve a high reliability. This new approach allows computing the intra-subject variability with only two measurements per subject, and predicting the reliability for a larger number of subjects and retests based on simulations, without requiring additional experiments. Such a tool of predictive value is especially valuable for target populations where time is scarce, e.g., for assessments in clinical settings.

## 1 Introduction

The development of assessments of human perception thresholds (e.g., visual, auditory, tactile, or proprioceptive stimuli) is a challenging field, as these require good psychometric and clinimetric properties such as high reliability, for both research and clinical applications. The selection and optimization of psychophysical assessments is, in general, a lengthy, iterative, and cumbersome process where different psychophysical methods need to be tested and their parameters tuned [1]. Evaluating such procedures requires time and financial resources, as it involves repeated assessment of a large number of subjects. This may present a serious hurdle for the development of reliable assessments, especially for sample populations where available time is scarce and recruitment is difficult or expensive (e.g., neurological patients).

When evaluating and optimizing psychophysical methods (e.g., for a high test-retest reliability), different factors play an essential role: method variability as well as inter- and intra-subject variability. While inter-subject variability clearly has an effect on reliability [2], it is given by the population of interest and its true value cannot be influenced. Previous works have suggested that a lack of correlation between different methods tested on the same subjects (i.e. a lack of agreement between results) may originate either from inherent method variability (i.e., based on the stochastic process, the statistical properties of the method, and number of trials) or from intra-subject variability [3]. As both method and intra-subject variability are confounded in the outcome measure of a perception assessment, it is difficult to discern one factor from the other and quantify them independently. Generalizability theory is one approach to disentangle different sources of errors [4–6]. However, it requires complex experimental designs with a large number of conditions where each factor is controlled for. Furthermore, unknown non-systematic and random error sources, such as the inaccuracy of the measurements and other uncontrolled factors (e.g., inattention or lack of motivation) remain confounded in the residual error.

The detection or discrimination capability of physical stimuli often resembles a sigmoidal psychometric function [7, 8]. This psychometric function defines the subject’s performance, or responses to physical stimuli in a psychophysical task. Therefore, perception and psychophysical procedures (i.e., complete perception experiments) can be modeled. As a matter of fact, the method variability as well as other performance metrics such as bias and efficiency can be quantified using computer simulations and have been widely investigated for various procedures [1, 9–20].

In contrast, intra-subject variability introduced by confounds (variables that influence both the dependent and independent variable causing a spurious association, e.g., inattention, lack of motivation in psychophysical experiments, or fatigue) is difficult to estimate and cannot be directly quantified based on experimental or simulated data only. For example, a lack of motivation could decrease the performance of a patient with sensory deficits in a perceptual test aiming to quantifying sensory deficits. Thus, it is not clear whether the origins of the decreased performance are the sensory deficits and/or lack of motivation. Because of such confounding effects, intra-subject variability has received little attention so far, and is generally neglected in computer simulations of psychophysical sampling procedures. As a consequence, simulations of psychophysical experiments are hardly realistic, and results are not representative.

The aim of this work is twofold: firstly, to present an approach to quantify intra-subject variability, and secondly, to apply and illustrate the approach by creating a general model of intra-subject variability—in this case of proprioceptive perception at the wrist assessed in a two-alternative forced-choice (2AFC) setting. To estimate the intra-subject variability for different parameters of the psychometric function, a dataset with repeated measures from a behavioral study is required. Based on this experimental data, the subject’s psychometric functions are modeled to simulate the same population. We propose to add individual, statistical noise distributions models on the different parameters (threshold and slope) of the psychometric functions to simulate intra-subject variability. The level of intra-subject variability (i.e., noise) on the different parameters can be quantified by matching the test-retest reliability of the simulated experiment with the test-retest reliability of the behavioral data and by maximizing the similarity between the distributions of outcome measures. Better knowledge about human perception and the ability to model intra-subject variability is important and would offer many possibilities, such as comparing, selecting, and tuning different psychophysical methods in simulated scenarios corresponding closely to the real application and population of interest. Furthermore, model-based extrapolation to a larger number of trials or increased sample size, for example to explore their impact on reliability, could then be performed purely in simulation. This could significantly speed up the development and testing of psychophysical assessment procedures.

## 2 Materials and methods

### 2.1 Behavioral data

#### 2.1.1 Subjects

Thirty-three healthy young subjects (*N*_subjects_ = 33) were recruited and participated in an experiment to assess wrist proprioception (age mean ± SD: 24.1 ± 3.4 years, 20 male and 13 female, 27 right handed, 5 left handed, and 1 ambidextrous). Handedness was assessed with the Edinburgh Handedness Inventory [21]. Exclusion criteria comprised sensory and motor deficits affecting normal wrist and hand function, as well as any history of neurological or wrist injury. Prior to participating in the experiment, all subjects gave written informed consent. The study was approved by the institutional ethics committee of the ETH Zurich (EK 2015-N-03).

#### 2.1.2 Protocol of the proprioceptive assessment

Each trial of the assessment aiming at estimating the difference threshold of the angular position at the right wrist joint consisted of the consecutive presentation of two different angles and the subsequent judgment by the subject which of the two presented movements was larger (two-interval 2AFC paradigm [8]). The subjects did not receive feedback about correct performance.

**Fig 1:**
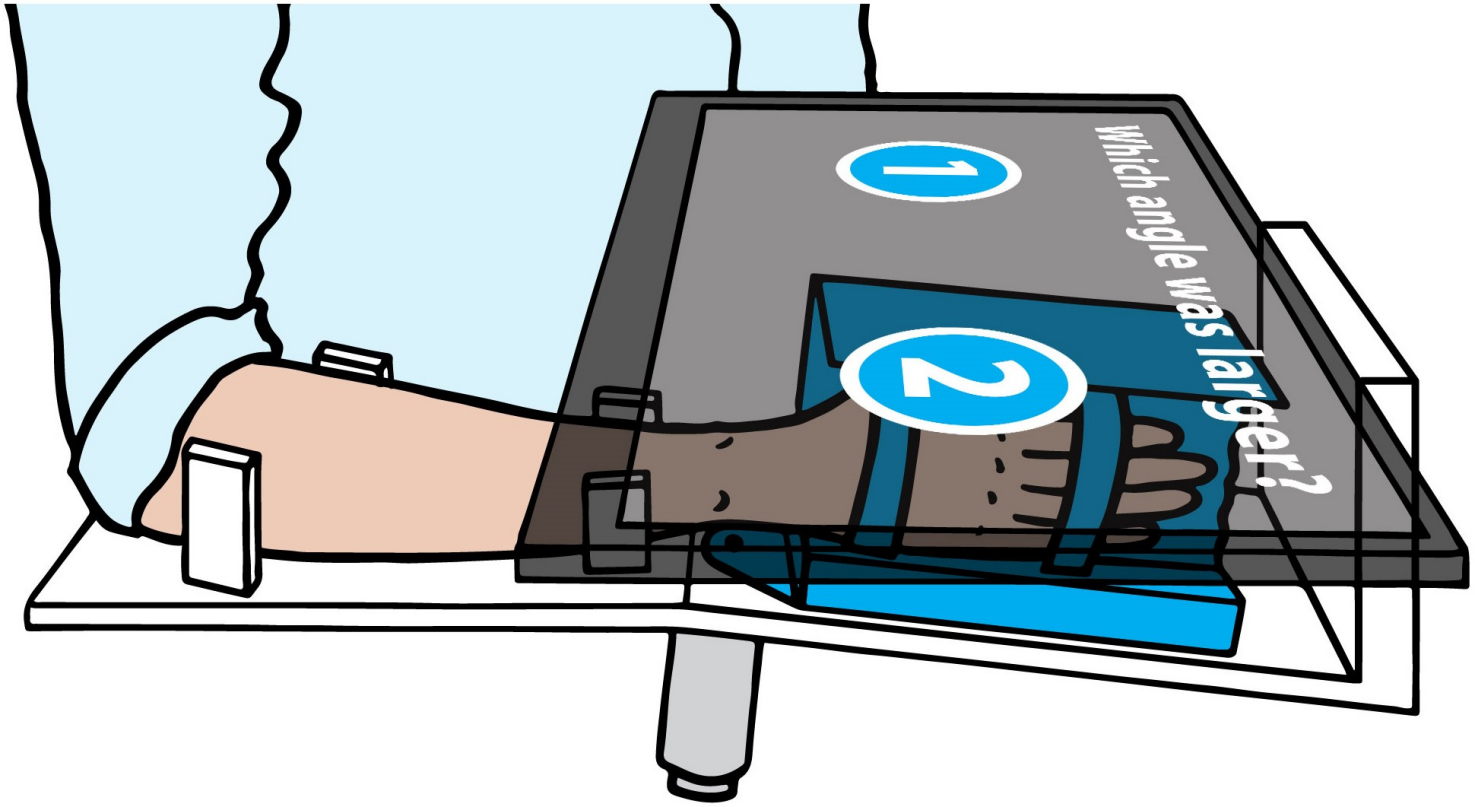
Robotic setup for the wrist proprioception assessments in the behavioral study. The motor (gray) actuates the handle (blue) in wrist flexion-extension direction. A touchscreen (semitransparent dark gray) placed over the wrist occludes the tested wrist from vision. With the non-assessed hand, the subject reports the response by clicking on one of the two blue buttons indicated on the screen.

The movements were applied to the passive wrist with a one degree-of-freedom robotic wrist interface (Fig 1). A detailed description of the robot can be found in [22]. In short, this device is capable of providing well-controlled and reproducible passive flexion-extension movements to the wrist and is driven by a direct-drive brushed DC motor (RE65, Maxon Motor, Sachseln, Switzerland). The angular position is measured with a high-resolution encoder (R158, 1 million counts/rev, Gurley Precision Instruments, Troy, NY, USA), and movements are controlled in LabVIEW RealTime 13.0 (National Instruments, Austin, TX, USA) at 1 kHz. Above the tested hand, a touchscreen showing the visual interface for the experiment is mounted horizontally. To avoid any visual or auditory cues (e.g., noise emitted by the motor), the tested arm was occluded from vision and white noise was played over headphones during the whole experiment.

The movements always started from the resting position (hand aligned with forearm, 0°) and went into flexion direction (maximum 40°). The two presented angles were always centered around a reference of 20°. The difference between the two angles (referred to as level) was defined by an adaptive sampling procedure named Parameter Estimation by Sequential Testing (PEST) [9]. PEST was used with a logarithmic adaptation for positive-only stimuli to avoid an undesired behavior of the algorithm due to zero crossings [3]. This adaptive algorithm takes the judgments (also referred to as responses) of past trials into account and changes the difference between the angles accordingly, using heuristic rules to approach the difference threshold as rapidly as possible. The same proprioceptive assessment has been previously used and described in more detail in other studies with a different robotic device for the assessment of the metacarpophalangeal joint [3, 23–25]. The same movement timing characteristics and parameters for the PEST algorithm were used in the present experiment, except for the maximum number of trials (start level *x*_0_ = 5.5°, start step Δ*x*_0_ = ±2°, target performance *P*_*t*_ = 75%, Wald sequential likelihood ratio test parameter *W* = 1, minimum step Δ*x*_min_ = ±0.1°, maximum trials at same level *trials*_max@*x*_ = 20, maximum trials in total *trials*_max_ = 120). Each flexion movement lasted 1 s and the wrist was kept at the wrist flexion angle for 1.5 s before moving back to the start position. Each movement followed a natural minimum jerk trajectory [26].

Each subject performed the assessment in five sessions on different days (from 1 to 4 days between sessions, with a maximum of 7 days from the first to the last session).

#### 2.1.3 Estimation of the psychometric function

**Fig 2:**
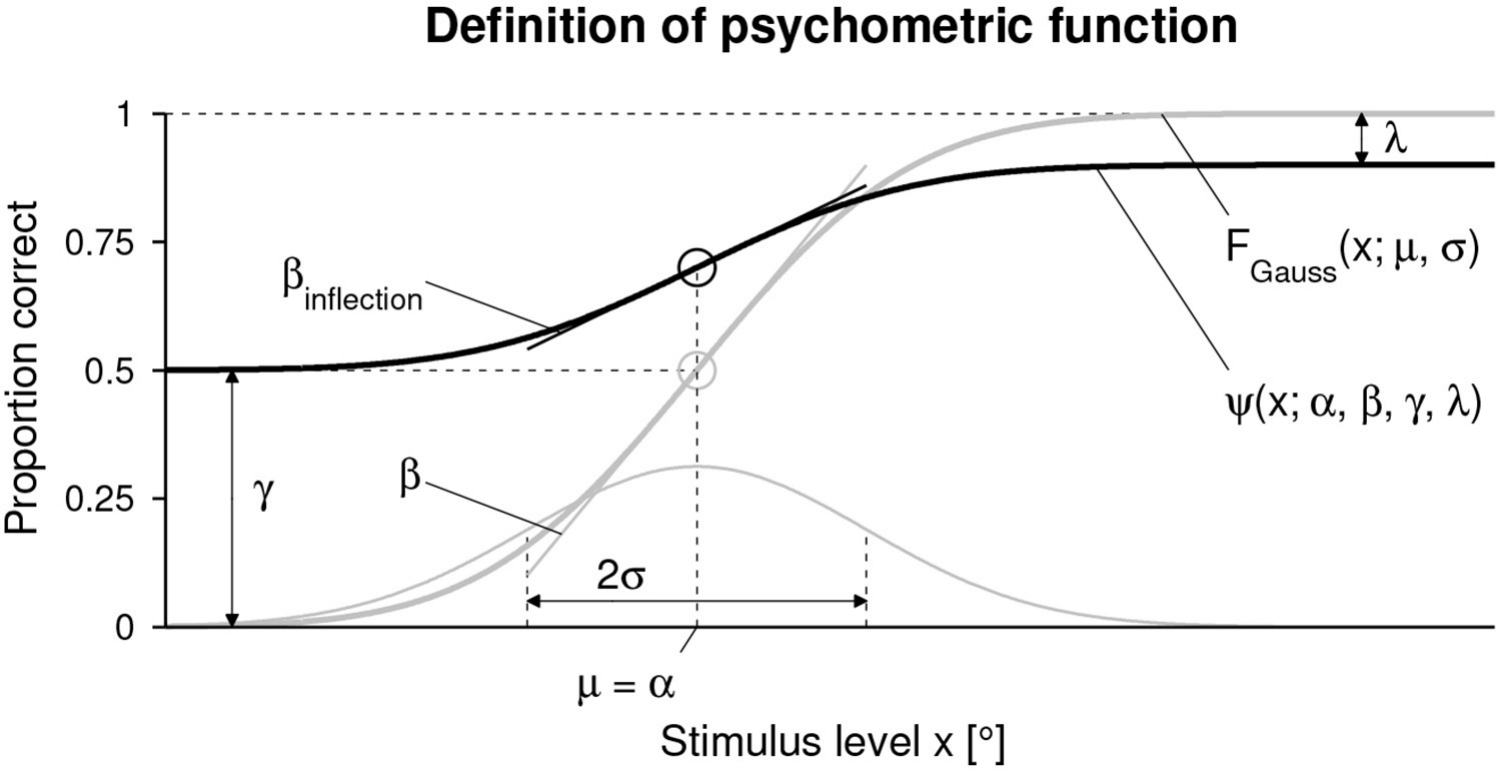
Definition of psychometric function and its parameters. Psychometric function *ψ*(*x*; *α*, *β*, *γ*, λ) (bold black sigmoid) and cumulative Gaussian function *F*_Gauss_(*x*; *μ*, *σ*) (bold gray sigmoid) in the case of a two-alternative forced choice (2AFC) task. The thin gray curve is the underlying Gaussian probability density function. The inflection points are indicated as circles in the respective colors.

Based on the data from the assessment sequence (i.e., difference between the two presented angles and corresponding response of the subject), the proportion of correct responses can be calculated for the different levels *x* to fit a sigmoidal psychometric function *ψ*(*x*) (Fig 2) using a Maximum Likelihood criterion implemented in the Palamedes MATLAB routines [27]:

$$\begin{array}{c} \psi (x;\alpha ,\beta ,\gamma ,\lambda )=\gamma +(1-\gamma -\lambda )F_{\text{Gauss}}(x;\mu ,\sigma^{2}), \end{array}$$ (1)

with *F*_Gauss_(*x*; *μ*, *σ*^2^) a sigmoidal cumulative Gaussian function. In the present work, the threshold parameter *α* corresponds to the mean *μ* of the underlying Gaussian function, and the slope parameter *β* is inversely proportional to the standard deviation *σ*:

$$\begin{array}{c} \beta =\frac{1}{\sqrt{2\pi }}\frac{1}{\sigma }. \end{array}$$ (2)

The guess rate parameter *γ* was fixed to 0.5, as the 2AFC paradigm with randomly ordered stimuli for the two intervals within a trial can be considered decision-bias-free, and the presentation of two identical stimuli should lead to performance at chance level [8]. The lapse rate parameter λ was allowed to vary ∈ [0, 0.1]. Leaving the lapse rate free when fitting a psychometric function has been shown to reduce estimation bias introduced by isolated scattered lapses [28]. Note that this range was chosen identical to our previous work [25] and is larger than the proposed range [0, 0.06] by [28]. This was motivated by the desire to account for a potentially higher probability of inattention in elderly or neurologically impaired subjects (for which such assessments are primarily designed), which was confirmed in [23].

The actual slope (first order derivative) of *ψ*(*x*) at the inflexion point *α* is

$$\begin{array}{c} \beta_{\text{inflection}}=\frac{\left(1-\gamma -\lambda \right)}{\sqrt{2\pi }}\frac{1}{\sigma }. \end{array}$$ (3)

This definition of the slope carries as units one over the units of the stimulus, in the present work [1/°], and can be used to compare the slope values across studies using different types of sigmoidal functions *F*(*x*) [29]. To do arithmetic calculations on the slope (e.g., arithmetic mean), it is reasonable to normalize the slope with the following nonlinear function to a range [0, 1] with arbitrary units [a.u.]:

$$\begin{array}{c} \beta_{\text{inflection}\;[\text{a}.\text{u}.]}=\frac{\arctan(\beta_{\text{inflection}})}{\pi /2}. \end{array}$$ (4)

If this nonlinear transformation is not performed, errors in slope estimation can diverge towards infinite for two almost identically steep psychometric functions, which would lead to a distortion when comparing to errors in shallow psychometric functions.

Using computer simulations, the estimation quality of psychophysical sampling procedures can be calculated by comparing the estimated values of the different parameters of a psychometric function with the true values (i.e., parameter values of the psychometric function to be estimated) [1]. Following this work, the estimation performance of PEST was evaluated with computer simulations using the same parameter values as used in the present behavioral study (*x*_0_ = 5.5°, Δ*x*_0_ = ±2°, *P*_*t*_ = 75%, *W* = 1, Δ*x*_min_ = ±0.1°, *trials*_max@*x*_ = 20, *trials*_max_ = 120). The variable error of the estimate cannot be corrected for. However, the average bias (i.e., also referred to as constant error of the estimate [1]) can be removed after fitting the psychometric function with the Maximum Likelihood criterion. While PEST can be considered a bias-free sampling procedure for the threshold estimates, the slope estimation bias showed a strong dependence on the true slope and was approximatively corrected by using the following equation:

$$\begin{array}{c} \beta_{\text{inflection,}\;\text{b}.\text{c}.\;[\text{a}.\text{u}.]}={\beta_{\text{inflection}\;[\text{a}.\text{u}.]}}^{2.381}. \end{array}$$ (5)

This power function (and the value of the exponent) are the result from a fit on simulated data from our previous work [1].

A further estimation bias in psychophysical experiments with human subjects can arise from longer inattention periods, as loss of attention may alter perception [30–32]. A method to detect and remove such inattention periods in PEST sequences has recently been proposed [25]. This method has shown to reduce estimation errors by up to around 75% and was applied *post-hoc* on the PEST sequences recorded in the behavioral study before fitting the psychometric function.

### 2.2 Computer simulations

#### 2.2.1 Population model and templates

A model of the population distribution was created for each parameter of the psychometric function (i.e., *α*, *β*, *γ*, and λ) based on the averaged parameters (across the five repeated measurements for each individual subjects) of the psychometric functions obtained in the behavioral study: For the threshold *α* and lapse rate λ, the arithmetic mean was calculated for each subject (across the five measurements) to obtain an improved estimate of the subject’s true psychometric function. The same was done for the slope *β*, however, *β* was first converted to the slope at inflection *β*_inflection_ (with the five corresponding lapse rates of the individual subject), normalized (*β*_inflection [a.u.]_), and the bias was removed (*β*_inflection, b.c. [a.u.]_) before averaging across the five measurements. Subsequently, the slope was converted back with the inverse transformations (with the averaged lapse of the individual subject). Averaging was not necessary for the guess rate *γ*, as it was always fixed to 0.5. Averaging over the repeated measurements was considered appropriate, as previous studies have shown with a mixed-effects model and a Bland-Altman analysis that there is no learning effect in this 2AFC proprioception assessment task [23, 24].

From these empirical parameter distributions a set of simulated perception models (also referred to as templates *ψ*(*x*)^*T*^) was randomly sampled. To differentiate between psychometric functions and their parameters originating from the behavioral study and the simulated psychometric functions, the symbol *T* was added for variables referring to simulated templates (e.g., *α*^*T*^). The number of templates *ψ*^*T*^(*x*) was set to be identical to the number of assessed subjects in the behavioral study (*N*_templates_ = 33).

#### 2.2.2 Noise model

When designing a noise model (i.e., continuous distribution) for a certain parameter of a psychometric function template (i.e., *α*^*T*^ or $\beta_{\text{inflection}\;[\text{a}.\text{u}.]}^{T}$), three things must be considered within this framework: (i) the continuous probability density function should be chosen based on an appropriate mathematical support dependent on the definition of the parameter, (ii) the mean *μ*_noise_ of the distribution should match the true parameter value of the template (i.e., *α*^*T*^ or $\beta_{\text{inflection}\;[\text{a}.\text{u}.]}^{T}$) for which the noise model is created, and (iii) the standard deviation of the noise model should be directly controllable by a noise level parameter (*ν*_*α*_ and *ν*_*β*_, respectively).

The threshold including intra-subject variability was modeled with a log–normal distribution with a support [0, +∞):

$$\begin{array}{c} \alpha_{\text{noise}}^{T}∼\text{Lognormal}\left(\mu_{N},\sigma_{N}^{2}\right). \end{array}$$ (6)

To avoid bias when introducing noise, the mean *μ*_noise_ was defined to be the threshold of the template:

$$\begin{array}{c} \mu_{\text{noise}}≔\alpha^{T}. \end{array}$$ (7)

The standard deviation *σ*_noise_ of the variability was controlled with the parameter *ν*_*α*_ ∈ [0, +∞):

$$\begin{array}{c} \sigma_{\text{noise}}≔\nu_{\alpha }, \end{array}$$ (8)

The two parameters of the log–normal distribution were calculated using *μ*_noise_ and the desired *σ*_noise_:

$$\begin{array}{c} \mu_{N}=\log(\frac{\mu_{\text{noise}}}{\sqrt{1+\frac{\sigma_{\text{noise}}^{2}}{\mu_{\text{noise}}^{2}}}})\;\text{and} \end{array}$$ (9)

$$\begin{array}{c} \sigma_{N}=\sqrt{\log(1+\frac{\sigma_{\text{noise}}^{2}}{\mu_{\text{noise}}^{2}})}. \end{array}$$ (10)

The slope including intra-subject variability was modeled with a beta distribution with a support [0, 1]:

$$\begin{array}{c} \beta_{\text{inflection}\;[\text{a}.\text{u}.],\;\text{noise}}^{T}∼Be\left(\alpha_{Be},\beta_{Be}\right). \end{array}$$ (11)

The mean *μ*_noise_ of $Be(\alpha_{Be},\beta_{Be})$ was defined to correspond to the normalized slope at the inflection of the template:

$$\begin{array}{c} \mu_{\text{noise}}≔\beta_{\text{inflection}\;[\text{a}.\text{u}.]}^{T}. \end{array}$$ (12)

The standard deviation *σ*_noise_ of the variability was controlled with the parameter *ν*_*β*_ ∈ (0, 1] serving as a scaling parameter:

$$\begin{array}{c} \sigma_{\text{noise}}≔\nu_{\beta }{\hat{\sigma }}_{\text{noise}}, \end{array}$$ (13)

where ${\hat{\sigma }}_{\text{noise}}$ is the maximum possible value for *σ*_noise_ to avoid a U-shaped distribution. This can be guaranteed with at least one of the parameters $\alpha_{Be}$ or $\beta_{Be}$ ≥ 1, leading to:

σ^noise=max(μnoise1-μnoise1+μnoiseμnoise(μnoise2-2μnoise+1)2-μnoise,μnoise(μnoise2-2μnoise+1)2-μnoise). (14)

With *μ*_noise_ and *σ*_noise_, the two parameters of the beta distribution $Be(\alpha_{Be},\beta_{Be})$ could be calculated:

$$\begin{array}{c} \alpha_{Be}=\frac{\mu_{\text{noise}}(-\mu_{\text{noise}}^{2}+\mu_{\text{noise}}-\sigma_{\text{noise}}^{2})}{\sigma_{\text{noise}}^{2}}\;\text{and} \end{array}$$ (15)

$$\begin{array}{c} \beta_{Be}=\alpha_{Be}(\frac{1-\mu_{\text{noise}}}{\mu_{\text{noise}}}). \end{array}$$ (16)

No noise was modeled on the lapse rate λ^*T*^ and on the guess rate *γ*^*T*^ = 0.5. The psychometric functions to be used for the simulated PEST sequences were of the form $\psi_{\text{noise}}^{T}\left(x;\alpha_{\text{noise}}^{T},\beta_{\text{noise}}^{T},\gamma^{T},\lambda^{T}\right)$. For the threshold, 16 equally distributed noise levels *ν*_*α*_ ∈ [0, 1.5], and for the slope, 14 noise levels *ν*_*β*_ ∈ [0, 1] with a twice as high grid density ∈ [0.7, 1], were simulated.

#### 2.2.3 Procedure

For each combination of *ν*_*α*_ and *ν*_*β*_, the PEST sequence of the 2AFC task was simulated five times for the whole set of templates $\Psi_{\nu_{\alpha },\nu_{\beta }}^{T}$. For each single simulated sequence, new random variables $\alpha_{\text{noise}}^{T}$ and $\beta_{\text{inflection}\;[\text{a}.\text{u}.],\;\text{noise}}^{T}$ were drawn from the log–normal and beta distributions, respectively, simulating intra-subject variability across the five measurements. The identical PEST parameters as in the behavioral study were used for the computer simulations. Responses to a specific level *x* were simulated by comparing a randomly generated number ∈U(0,1) to $\psi_{\text{noise}}^{T}\left(x\right)$ of the respective template. A smaller random number generated a correct response, and a larger random number a false response.

The simulation of the whole set $\Psi_{\nu_{\alpha },\nu_{\beta }}^{T}$ was repeated *N*_simulations_ = 1000 times for each combination of *ν*_*α*_ and *ν*_*β*_ with new randomly sampled parameters (i.e., *α*^*T*^, *β*^*T*^, *γ*^*T*^, λ^*T*^) from the population distribution models.

The psychometric functions from the simulated PEST sequences were estimated identically to the behavioral study, including the bias correction. The only difference lay in the inattention correction algorithm [25], which was not applied on the simulated data. It was assumed that significant biases from potential inattention periods in the behavioral study were already corrected for before creating the population model for the templates. Thus, as no inattention periods were modeled in the simulations, there was no need to apply the algorithm. The computer simulations and estimations of the psychometric function were performed entirely in MATLAB R2014a.

### 2.3 Data analysis

Test-retest reliability of the estimated thresholds from the five measurements of the behavioral study was quantified by computing the ICC(2,1) intraclass correlation coefficient *r* (two-way layout with random effects for absolute agreement) [33] and its 95% confidence interval (CI) [34, 35].

Identically, for each set $\Psi_{\nu_{\alpha },\nu_{\beta }}^{T}$, distributions of *N*_simulations_ values for the reliability of the estimated thresholds as well as its lower and upper CI bounds for each combination of *ν*_*α*_ and *ν*_*β*_ were generated. From these *N*_simulations_ reliability values, the arithmetic mean ${\bar{r}}_{\nu_{\alpha },\nu_{\beta }}^{T}$ was calculated. In this two-dimensional noise space an iso-reliability contour where the reliability of the simulated experiment matched the reliability of the behavioral study (${\bar{r}}_{\nu_{\alpha },\nu_{\beta }}^{T}=r$) was calculated (set of *ν*_*α*_ and *ν*_*β*_ pairs). To obtain a smoother contour, the reliability surface was interpolated with a spline on a finer grid (by halving the grid intervals three times in each dimension).

To find which *ν*_*α*_ and *ν*_*β*_ pair of the iso-reliability contour corresponds the best to the intra-subject variability of the behavioral study, for each of the *N*_simulations_ per pair, histograms of the parameters of the estimated psychometric functions from the computer simulation were compared to histograms of the parameters of the psychometric functions originating from the behavioral data. This was done by calculating the cosine similarity between the two vectors of histogram bin counts (**h** and **h**^*T*^, for the behavioral and simulated data, respectively) for the parameters *α*, *β*_inflection, b.c. [a.u.]_, and λ:

$$\begin{array}{c} similarity_{i}^{T}=\cos\left(\theta_{i}\right)=\frac{h_{i}\cdot h_{i}^{T}}{∥h_{i}{∥}_{2}∥h_{i}^{T}{∥}_{2}}\;\forall i\in \left\{\alpha ,\beta ,\lambda \right\}, \end{array}$$ (17)

where a similarity of 1 represents identical histograms. Note that by using this similarity metric the histograms do not need to be additionally normalized. The following bin sizes were used for *α*, *β*_inflection, b.c. [a.u.]_, and λ: 0.25, 0.05, and 0.005. To obtain an overall similarity, the three calculated similarities were multiplied with each other.

$$\begin{array}{c} s_{\nu_{\alpha },\nu_{\beta }}^{T}=\prod_{i\in \{\alpha ,\beta ,\lambda \}}similarity_{i}^{T}, \end{array}$$ (18)

From these *N*_simulations_ overall similarity values, the arithmetic mean ${\bar{s}}_{\nu_{\alpha },\nu_{\beta }}^{T}$ was calculated. The iso-reliability contour was projected onto the similarity surface in the two-dimensional noise space after a spline interpolation, identical to what was done for the reliability. The interpolated *ν*_*α*_ and *ν*_*β*_ pair on the iso-reliability contour with the highest average overall similarity was chosen as the best model to estimate intra-subject variability (${\hat{\nu }}_{\alpha }$ and ${\hat{\nu }}_{\beta }$).

For a new set of psychometric functions $\Psi_{{\hat{\nu }}_{\alpha },{\hat{\nu }}_{\beta }}^{T}$ with the optimal noise model, the simulation was repeated *N*_simulations_ times, and the parameter distributions as well as ${\bar{r}}_{{\hat{\nu }}_{\alpha },{\hat{\nu }}_{\beta }}^{T}$ and ${\bar{s}}_{{\hat{\nu }}_{\alpha },{\hat{\nu }}_{\beta }}^{T}$ were calculated. In addition, the maximum attainable reliability ${\hat{r}}_{{\hat{\nu }}_{\alpha },{\hat{\nu }}_{\beta }}^{T}$ (corresponding to no method variability) was computed based directly on the templates with intra-subject noise, but without simulating the psychophysical experiment.

To illustrate the intra-subject variability on a psychometric function, a population average model was computed by averaging the individual subject models. Using the intra-subject variability models with parameters ${\hat{\nu }}_{\alpha }$ and ${\hat{\nu }}_{\beta }$, 1000 templates were created. The estimate distributions originating from pure method variability as well as from intra-subject variability were compared with each other by plotting the percentage of estimates within a tolerance interval depending on the interval size (percentage within bounds, *PCTw*/*iB*), and the normalized area under these curves (*nAUC*) according to the methods proposed by [1].

## 3 Results

**Fig 3:**
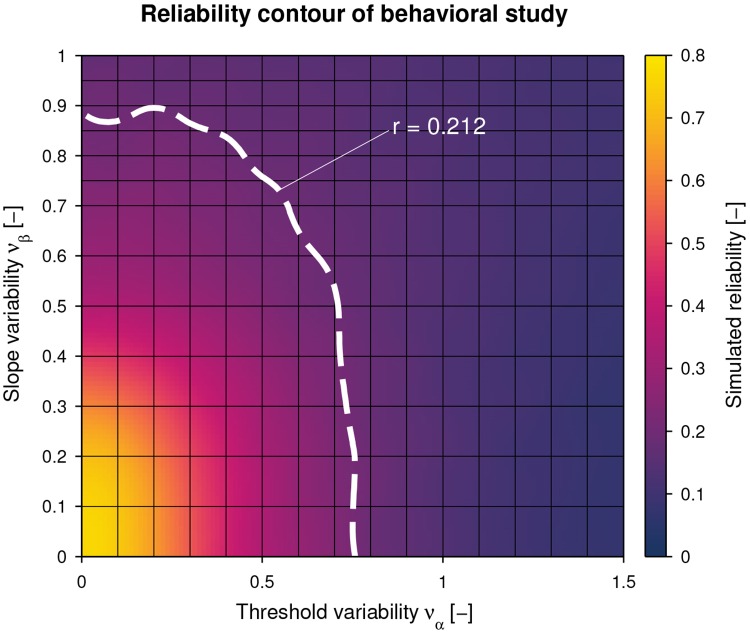
Simulated reliability and iso-reliability contour of behavioral study. For each pair of intra-subject threshold noise *ν*_*α*_ and slope noise *ν*_*β*_, the simulated reliability averaged across *N*_simulations_ = 1000 simulations (${\bar{r}}_{\nu_{\alpha },\nu_{\beta }}^{T}$) is represented as a heat map. The dashed white line indicates the iso-reliability contour corresponding to the reliability obtained from the behavioral study (*r* = 0.212).

The test-retest reliability coefficient of the behavioral study and its confidence interval was *r* = 0.212 [0.077, 0.394]. The simulated reliability ${\bar{r}}_{\nu_{\alpha },\nu_{\beta }}^{T}$ for different *ν*_*α*_ and *ν*_*β*_ pairs as well as the matched iso-reliability contour at *r* are shown in Fig 3. In case of no intra-subject variability, the reliability would correspond to ${\bar{r}}_{\nu_{\alpha }=0,\nu_{\beta }=0}^{T}=0.768$ [0.662, 0.859] for the psychophysical paradigm and sampling procedure used in this work (i.e., maximum attainable reliability using these methods for the present population of interest).

**Fig 4:**
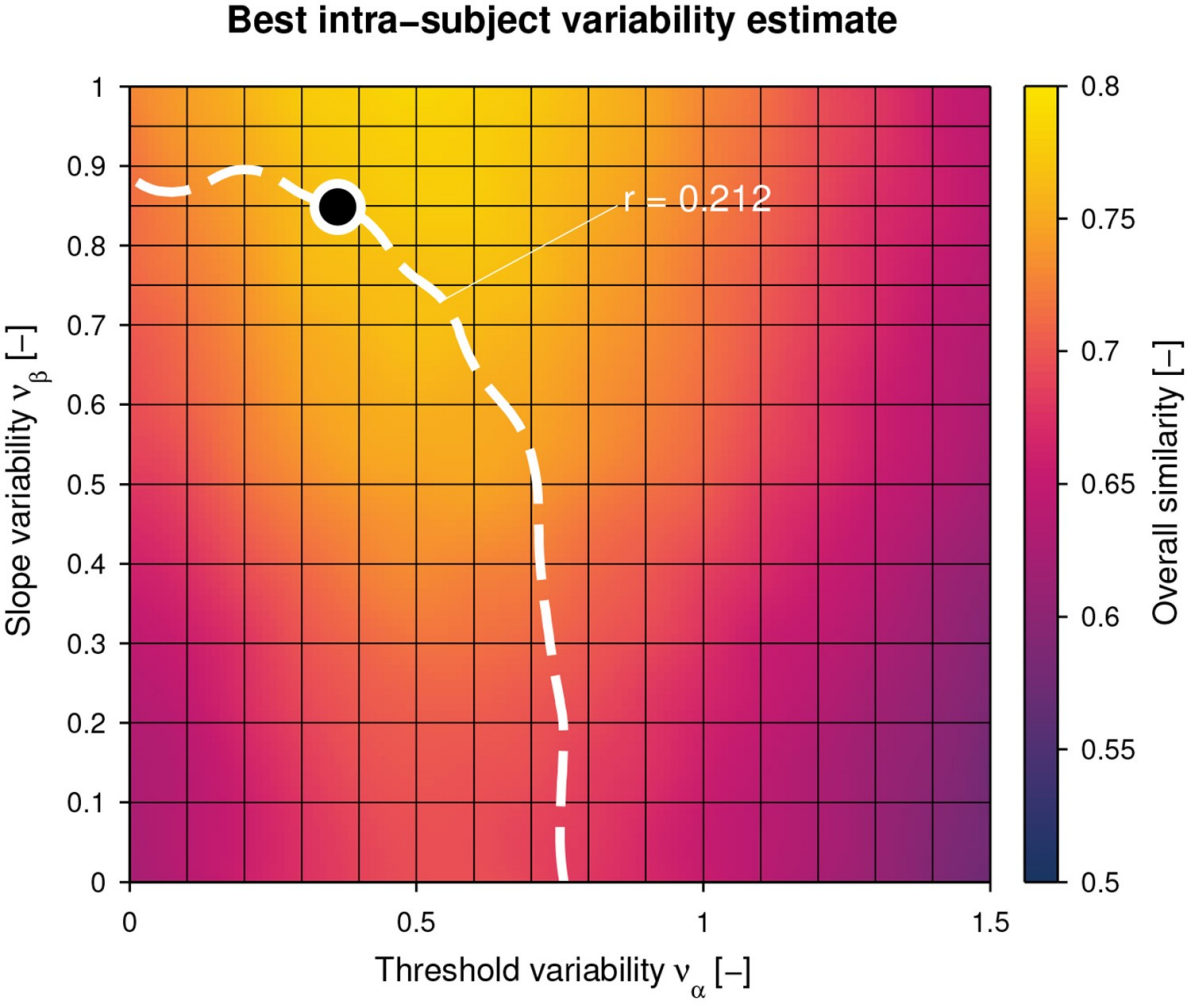
Best intra-subject variability estimate based on overall similarity. For each pair of intra-subject threshold noise *ν*_*α*_ and slope noise *ν*_*β*_, the overall similarity (combined for threshold, slope, and lapse rate) averaged across *N*_simulations_ = 1000 simulations (${\bar{s}}_{\nu_{\alpha },\nu_{\beta }}^{T}$) is represented as a heat map. The dashed white iso-reliability contour is identical to Fig 3. The noise level pair on the contour with the highest overall similarity (${\bar{s}}_{{\hat{\nu }}_{\alpha },{\hat{\nu }}_{\beta }}^{T}=0.764$) is indicated with a black dot.

**Fig 5:**
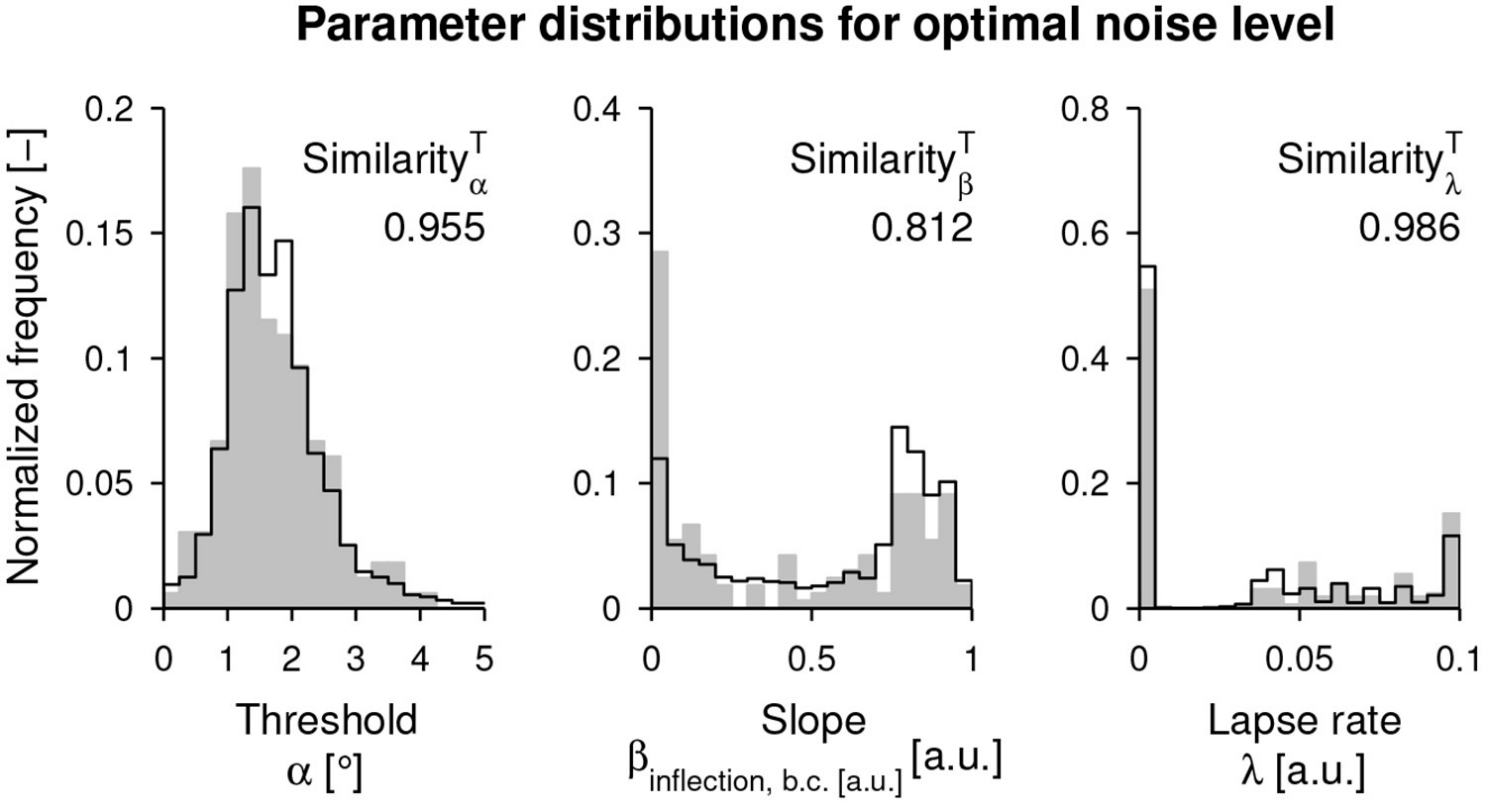
Histogram similarity for the optimal intra-subject variability. Histograms of the parameters of the psychometric functions of the behavioral data (gray fill, 33 × 5 data points) versus the simulated data (black outline, 33 × 5 simulated data points averaged over 1000 simulations) with optimal noise level at the pair ${\hat{\nu }}_{\alpha }$, ${\hat{\nu }}_{\beta }$.

The overall similarity ${\bar{s}}_{\nu_{\alpha },\nu_{\beta }}^{T}$ (combined for threshold, slope, and lapse rate) is visualized in Fig 4, together with the same projected iso-reliability contour. The maximum overall similarity on the contour was found for the noise level pair ${\hat{\nu }}_{\alpha }=0.363$ and ${\hat{\nu }}_{\beta }=0.849$ (${\bar{s}}_{{\hat{\nu }}_{\alpha },{\hat{\nu }}_{\beta }}^{T}=0.764$), corresponding to the best intra-subject variability model estimate. The similarities of the distributions of the parameters of the psychometric functions are shown individually in Fig 5. The simulated reliability at this noise level pair was ${\bar{r}}_{{\hat{\nu }}_{\alpha },{\hat{\nu }}_{\beta }}^{T}=0.207$ [0.076, 0.384]. The maximum attainable reliability without method variability (i.e., assuming a perfect assessment) for the identified intra-subject variability model would be ${\hat{r}}_{{\hat{\nu }}_{\alpha },{\hat{\nu }}_{\beta }}^{T}=0.552$ [0.403, 0.704].

**Fig 6:**
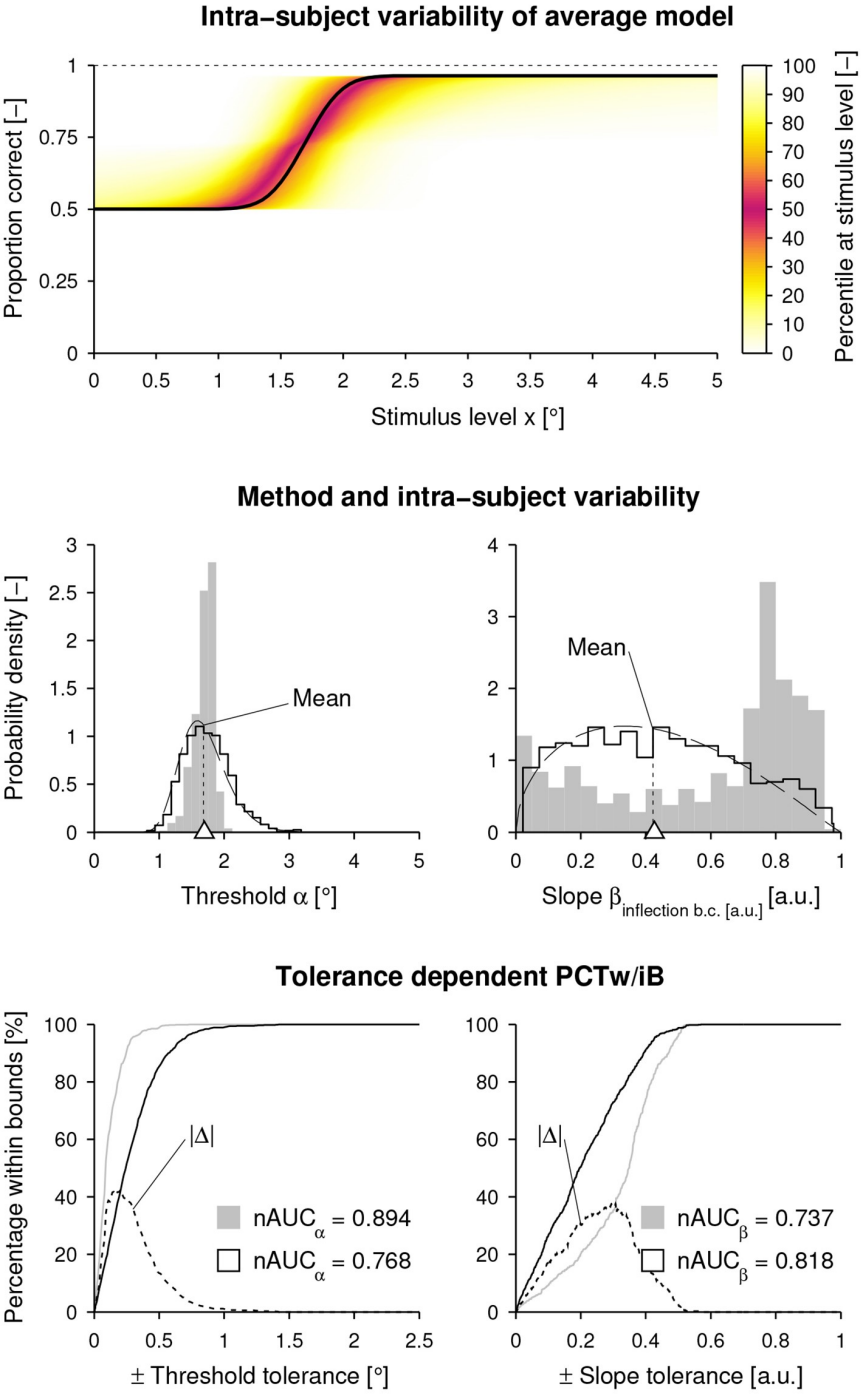
Illustration of intra-subject variability. **(Top)** For each stimulus level *x* the distribution of proportion correct is plotted as a heat map based on 1000 templates created with the population average model (bold black sigmoid) and the intra-subject variability models using ${\hat{\nu }}_{\alpha }$ and ${\hat{\nu }}_{\beta }$. **(Middle)** The dashed black distribution curves correspond to the parametric log–normal and beta intra-subject variability models. The histograms (black outline) as well as the dashed black lines for the means show the parameter distributions of the 1000 templates including intra-subject variability. The white triangles indicate the threshold and slope of the population average model (without noise). As a comparison, the inherent method variability (histogram with gray fill) for the same population average model without intra-subject variability is plotted. **(Bottom)** The percentage of estimates within a tolerance interval (percentage within bounds, *PCTw*/*iB*) around the parameters of the population average model is plotted against the size of the interval (gray: method variability, black: intra-subject variability), together with the absolute difference of percentage (|Δ|, dashed black line). For both method and intra-subject variability, the normalized area under the curve (*nAUC*) is calculated.

For illustration purposes, the effect of intra-subject variability on the shape of the psychometric function is shown in Fig 6 for the population average model *ψ*(*x*; *α* = 1.696, *β* = 1.708, *γ* = 0.500, λ = 0.036) and the noise level pair ${\hat{\nu }}_{\alpha }$, ${\hat{\nu }}_{\beta }$, together with the distributions of threshold and slope resulting from method and intra-subject variability. For the threshold estimation, the *nAUC* was higher for the method variability compared to the intra-subject variability, whereas for the slope estimation, the opposite was the case. The maximum difference in estimation performance in terms of *PCTw*/*iB* was 42.5% at a threshold tolerance of ±0.210°, and 38.1% at a slope tolerance of ±0.299.

## 4 Discussion

In this work we presented an approach to quantify intra-subject variability in psychophysical testing. This was achieved by introducing and adjusting a statistical noise model in computer simulations to match the test-retest reliability and histograms of the parameters of the estimated psychometric functions of a behavioral dataset. Using this approach we estimated the intra-subject variability of healthy subjects in a psychophysical assessment of proprioceptive perception at the wrist using a 2AFC paradigm, and compared the intra-subject variability with the inherent method variability of PEST.

The results showed that for a matched reliability, the similarity between the behavioral and simulated datasets was excellent for the optimal pair of intra-subject threshold and slope variability. Furthermore, the identified intra-subject variability of the threshold was larger compared to the method variability, whereas the opposite was the case for the slope.

### 4.1 Intra-subject and method variability

When trying to estimate the test-retest reliability based on the population model without intra-subject variability, the reliability coefficient would be largely overestimated. In the present sample population this would result in a considerable error of 262.3%. In contrast, when including intra-subject variability in the simulation, the reliability of the simulated experiment matched the reliability of the behavioral study with an absolute error of 0.005 (${\bar{r}}_{{\hat{\nu }}_{\alpha },{\hat{\nu }}_{\beta }}^{T}=0.207$ and *r* = 0.212, respectively), corresponding to a relative error of 2.4%. In theory, this error should be zero, however, since the estimates were based on a stochastic generation of responses, the simulated test-retest reliability varied across simulation runs. To improve the estimate of intra-subject variability, and therefore the match of reliability values, a high number of repetitions (*N*_simulations_) were performed to obtain higher statistical power, and the grid of the simulated intra-subject variability levels in the two-dimensional reliability space was interpolated. This error could be further minimized by increasing the number of repetitions and the density of the simulation grid. Further indication for a good model estimation quality is provided by the fact that not only the simulated and behavioral reliability coefficient matched, but also matching errors for the CI were low (absolute [0.001, 0.010] and relative [1.3%, 2.5%] errors for the lower and upper bound). Moreover, cosine similarity between behavioral and simulated outcome measures was very high for all three parameters *α*, *β*, and λ (> 0.8), and thus demonstrates that the population’s inter- and intra-subject variability models accurately represent the actual population.

The presented method allows to discern between and compare intra-subject variability and method variability. When assuming invariant subjects (i.e., no intra-subject variability), the test-retest reliability for the threshold would be 39.2% higher compared to when the estimated intra-subject variability is included in the simulation, but a perfect method (i.e., no method variability) would be assumed. This is also reflected by the *nAUC* for the threshold (a non-parametric metric to evaluate the variability of estimation errors), which is higher by 16.4% for the simulated case with method variability only. Based on these findings, if the assessment was to be improved, one could suggest to address factors influencing the intra-subject variability, before optimizing the psychophysical sampling procedure, as even with a perfect method, the reliability would ceil at ${\hat{r}}_{{\hat{\nu }}_{\alpha },{\hat{\nu }}_{\beta }}^{T}=0.552$ due to intra-subject variability. This can also be seen in Fig 6 **(Middle, left)** where the distribution of estimates is narrower for the method variability compared to the one for intra-subject variability. It can also be observed that the method variability follows a unimodal distribution, resembling a log–normal probability density function, as it can be expected from the theoretical definition of the threshold parameter with positive semi-infinite support. On the contrary, the slope estimates suffer from poor method performance and, according to the U-shaped estimate distribution (histogram with gray fill in Fig 6 **(Middle, right)**), outcome measures are predominantly severely under- or overestimated. This poor slope estimation performance given the settings of the sampling procedure and the short number of trials has also been observed in [1]. As a consequence, the *nAUC* for the simulated case with intra-subject variability only is 11% higher. Thus, if the slope estimation should be improved, it would be important to optimize the current sampling procedure or choose another sampling procedure (e.g., the *Ψ* method, designed to estimate both the threshold and the slope [36]).

### 4.2 Advantages and limitations of this method

The advantage of this method is that the test-retest reliability is affected by all terms of variability (inter- and intra-subject, and method variability). As a consequence, since the inter-subject variability can be approximated by taking the averaged psychometric functions for each subject and the method variability is given by the simulation, the intra-subject variability can be estimated. Furthermore, the intra-subject variability can be calculated even if only two measurements were done per subject, whereas, for example, calculating the standard deviation of two measurements for each subject is very likely a poor estimate of the true intra-subject variability (besides being still confound with method variability). However, it should be noted that, depending on the intra-subject and method variability, the quality of the model of the population (and inter-subject variability) can be compromised if only two measurements are available per subject. Thus, in case of a poor population model, an overestimated inter-subject variability may be compensated by an underestimated intra-subject variability and vice versa when matching the reliability. Ideally, the available behavioral data would encompass a large sample size (for a good representation of the population) and a large number of measurements (for a good estimate of each subject’s psychophysical function). An advantage of sampling templates from the computed distributions representing the population compared to using the averaged psychometric functions as templates, is that repeated randomly sampled templates should lead to more generalizable results than bootstrapping from a limited set of subjects. More importantly, it offers the possibility to sample more templates from the distribution, for example to predict how the reliability and its confidence interval changes with increasing sample size. This framework can be applied to any psychophysical assessment, where the performance of the subject can be modeled. However, it should be noted that dedicated behavioral data and simulations are required for every individual application. Within the same application, the estimated models can be used to extrapolate, e.g., to larger sample sizes. Transferability of the intra-subject variability model from one population to another (e.g., from healthy subjects to neurologically impaired patients) might be limited and subject to further investigation. Nevertheless, it can be assumed that using the intra-subject variability model for healthy subjects in a simulation of a patient population provides better reliability estimates than having no intra-subject variability model included. Furthermore, if it can be assumed that the psychophysical sampling procedure (e.g., PEST) does not strongly influence confounds or affect the intra-subject variability, the same models could be used to create realistic simulations to compare different sampling procedures.

A limitation of the present simulations is that no intra-subject variability was modeled for the lapse rate. It would be possible model the lapse rate including intra-subject variability with a beta distribution as for the slope, but with an adapted support. However, for the sake of simplicity, this was omitted here. As a matter of fact, as the histogram similarity is almost 1 for the lapse rate parameter, it shows that using a constant lapse rate (within the range [0, 0.1]) for each individual subject also leads to realistic simulations and that adding an additional intra-subject variability model for the lapse rate may not be necessary. While Fig 5 suggests that there may be cases with lapse rates higher than 0.1, this would correspond to subjects not paying attention to every fifth trial, which is very high for healthy subjects. More likely, this bimodal result (lapse rates around 0 and around 0.1) may arise from the fitting procedure and the short number of trials per PEST sequence. To identify the origin of these results, further studies comparing different fitting procedures with longer sequences would be required. When identifying the best model of intra-subject variability, the noise level pair *ν*_*α*_, *ν*_*β*_, where overall similarity is the highest, may not lie on the iso-reliability contour corresponding to the reliability *r* of the behavioral data. One reason for this is that in the similarity histogram, inter- and intra-subject variability are confounded, and the similarity may vary depending on the selection of bin sizes. In contrast, using the reliability as a metric should provide a more robust and accurate estimate of the variability model, as it distinguishes between inter- and intra-subject variability despite taking both into account. Therefore, the overall similarity is used only as a second criterion to identify the optimal model. One major limitation of this approach to estimate intra-subject variability is that it only provides one variability model for the whole sample and not individual models for each subject. To create individual models, more repeated measurements would be necessary for each subject. However, the present noise models are already a significant improvement over no variability model, and may be accurate enough for many applications.

## 5 Conclusions

Computer simulations offer a valuable and powerful tool to simulate and optimize psychophysical experiments. While they can be used to evaluate different procedures and their method variability, existing computer simulations are often not representative of real-world scenarios, as critical aspects such as the intra-subject variability are neglected. As a matter of fact, intra-subject variability cannot be directly quantified from behavioral data. This work introduces a new approach based on the combination of computer simulations and behavioral data to separate method variability from intra-subject variability and to estimate and model intra-subject variability in psychophysical experiments.

Given a realistic model of the population, different psychophysical procedures can be simulated and compared, and the procedures can be tuned to the specific application and target population. Quantifying the method and intra-subject variability allows putting them into perspective when developing assessments. Given the intra-subject variability, it allows simulating an experiment with an ideal psychophysical method (i.e., finding the theoretically maximally attainable performance of an assessment). These two aspects can inform the decision whether effort should be spent on improving the psychophysical procedure (i.e., reducing method variability) or if potential confounds affecting intra-subject variability should be addressed. The efficiency of attempts to reduce confounds (e.g., inattention [25]) could be quantified (using the presented method) based on a reduction of the intra-subject variability. Furthermore, based on the more complete model also containing intra-subject variability, it is also possible to examine the impact of a larger number of trials on reliability, or the converging behavior of the reliability’s confidence interval bounds with a larger number of subjects, as well as retests, without having to conduct additional experiments. This presents a particular benefit for studies with populations where time for assessments is limited or expensive, as in the case of a clinical setting.

## Supporting information

S1 File Behavioral data. This file contains three tables with the threshold, slope at inflection, and lapse rate obtained in the behavioral study. The columns are subject ID and measurements 1 through 5.
(XLSX)

## References

[1] Rinderknecht MD, Lambercy O, Gassert R. Performance Metrics for an Application-driven Selection and Optimization of Psychophysical Sampling Procedures. PLOS ONE. 2018;13(11). 10.1371/journal.pone.0207217 30485350 PMC6261547

[2] Streiner DL, Norman GR. Health measurement scales: a practical guide to their development and use. USA: Oxford university press; 2008.

[3] Rinderknecht MD, Popp WL, Lambercy O, Gassert R. Experimental Validation of a Rapid, Adaptive Robotic Assessment of the MCP Joint Angle Difference Threshold. In: Auvray M, Duriez C, editors. Haptics: Neuroscience, Devices, Modeling, and Applications. Lecture Notes in Computer Science. Berlin, Heidelberg: Springer Berlin Heidelberg; 2014. p. 3–10.

[4] Atkinson G, Nevill AM. Statistical Methods For Assessing Measurement Error (Reliability) in Variables Relevant to Sports Medicine. Sports Medicine. 1998;26(4):217–238. 10.2165/00007256-199826040-00002 9820922

[5] Brennan RL. Generalizability Theory. Educational Measurement: Issues and Practice. 1992;11(4):27–34. 10.1111/j.1745-3992.1992.tb00260.x

[6] Roebroeck ME, Harlaar J, Lankhorst GJ. The Application of Generalizability Theory to Reliability Assessment: An Illustration Using Isometric Force Measurements. Physical Therapy. 1993;73(6):386–395. 10.1093/ptj/73.6.386 8497513

[7] Gescheider G. Psychophysics: The Fundamentals. New Jersey: Lawrence Erlbaum Associates; 1997.

[8] Macmillan NA, Douglas Creelman C. Detection Theory: A User’s Guide. New Jersey: Lawrence Erlbaum Associates; 2005.

[9] Taylor MM, Douglas Creelman C. PEST: Efficient estimates on probability functions. The Journal of the Acoustical Society of America. 1967;41:782 10.1121/1.1910407

[10] Taylor MM. On the efficiency of psychophysical measurement. The Journal of the Acoustical Society of America. 1971;49(2):Suppl 2:505–Suppl 2:508. 10.1121/1.1912379 5541746

[11] Findlay J. Estimates on probability functions: A more virulent PEST. Attention, Perception, & Psychophysics. 1978;23:181–185. 10.3758/BF03208300

[12] Pentland A. Maximum likelihood estimation: The best PEST. Attention, Perception, & Psychophysics. 1980;28(4):377–379. 10.3758/BF03204398 7465322

[13] Hall JL. Hybrid adaptive procedure for estimation of psychometric functions. The Journal of the Acoustical Society of America. 1981;69:1763 10.1121/1.385912 7240589

[14] Madigan R, Williams D. Maximum-likelihood psychometric procedures in two-alternative forced-choice: evaluation and recommendations. Perception & Psychophysics. 1987;42(3):240–249. 10.3758/BF03203075 3671049

[15] Simpson WA. The step method: A new adaptive psychophysical procedure. Perception & Psychophysics. 1989;45(6):572–576. 10.3758/BF03208065 2740198

[16] Watson AB, Fitzhugh A. The method of constant stimuli is inefficient. Perception & Psychophysics. 1990;47(1):87–91. 10.3758/BF03208169 2300429

[17] Kaernbach C. Simple adaptive testing with the weighted up-down method. Perception & Psychophysics. 1991;49(3):227–229. 10.3758/BF03214307 2011460

[18] Green DM. A maximum-likelihood method for estimating thresholds in a yes-no task. The Journal of the Acoustical Society of America. 1993;93(4):2096–2105. 10.1121/1.406696 8473622

[19] King-Smith PE, Grigsby SS, Vingrys AJ, Benes SC, Supowit A. Efficient and unbiased modifications of the QUEST threshold method: theory, simulations, experimental evaluation and practical implementation. Vision Research. 1994;34(7):885–912. 10.1016/0042-6989(94)90039-6 8160402

[20] Faes L, Nollo G, Ravelli F, Ricci L, Vescovi M, Turatto M, Pavani F, Antolini R. Small-sample characterization of stochastic approximation staircases in forced-choice adaptive threshold estimation. Perception & Psychophysics. 2007;69(2):254–262. 10.3758/BF03193747 17557595

[21] Oldfield RC. The assessment and analysis of handedness: the Edinburgh inventory. Neuropsychologia. 1971;9(1):97–113. 10.1016/0028-3932(71)90067-4 5146491

[22] Chapuis D, De Grave RB, Lambercy O, Gassert R. ReFlex, a haptic wrist interface for motor learning and rehabilitation. In: Haptics Symposium, 2010 IEEE. Waltham, Massachusetts, USA: IEEE; 2010. p. 417–424.

[25] Rinderknecht MD, Lambercy O, Raible V, Büsching I, Sehle A, Liepert J, Gassert R. Reliability, validity, and clinical feasibility of a rapid and objective assessment of post-stroke deficits in hand proprioception. Journal of NeuroEngineering and Rehabilitation. 2018;15(1). 10.1186/s12984-018-0387-6 29880003 PMC5991441

[23] Rinderknecht MD, Lambercy O, Raible V, Liepert J, Gassert R. Age-based model for metacarpophalangeal joint proprioception in elderly. Clin Interv Aging. 2017;12:635–643. 10.2147/CIA.S129601 28435235 PMC5388205

[25] Rinderknecht MD, Ranzani R, Popp WL, Lambercy O, Gassert R. Algorithm for improving psychophysical threshold estimates by detecting sustained inattention in experiments using PEST. Attention, Perception, & Psychophysics. 2018;80(6):1629–1645. 10.3758/s13414-018-1521-z 29748784

[26] Hogan N. Adaptive control of mechanical impedance by coactivation of antagonist muscles. Autom Control, IEEE Trans. 1984;29(8):681–90. 10.1109/TAC.1984.1103644

[27] Prins N, Kingdom FAA. Palamedes: Matlab routines for analyzing psychophysical data.; 2009. Available from: http://www.palamedestoolbox.org.

[28] Wichmann FA, Hill NJ. The psychometric function: I. Fitting, sampling, and goodness of fit. Perception & Psychophysics. 2001;63(8):1293–1313. 10.3758/BF03194544 11800458

[29] Strasburger H. Converting between measures of slope of the psychometric function. Perception & Psychophysics. 2001;63(8):1348–1355. 10.3758/BF03194547 11800461

[30] Leek MR, Hanna TE, Marshall L. An interleaved tracking procedure to monitor unstable psychometric functions. The Journal of the Acoustical Society of America. 1991;90(3):1385–1397. 10.1121/1.401930 1939903

[31] Fründ I, Haenel NV, Wichmann FA. Inference for psychometric functions in the presence of nonstationary behavior. J Vis. 2011;11(6). 10.1167/11.6.16 21606382

[32] Cohen MR, Maunsell JHR. When attention wanders: how uncontrolled fluctuations in attention affect performance. J Neurosci. 2011;31(44):15802–15806. 10.1523/JNEUROSCI.3063-11.2011 22049423 PMC3579494

[33] Shrout PE, Fleiss JL. Intraclass correlations: uses in assessing rater reliability. Psychol Bull. 1979;86(2):420–428. 10.1037/0033-2909.86.2.420 18839484

[34] Lexell JE, Downham DY. How to assess the reliability of measurements in rehabilitation. Am J Phys Med Rehabil. 2005;84(9):719–723. 10.1097/01.phm.0000176452.17771.20 16141752

[35] de Vet HCW, Terwee CB, Knol DL, Bouter LM. When to use agreement versus reliability measures. J Clin Epidemiol. 2006;59(10):1033–1039. 10.1016/j.jclinepi.2005.10.015 16980142

[36] Kontsevich LL, Tyler CW. Bayesian adaptive estimation of psychometric slope and threshold. Vision Research. 1999;39(16):2729–2737. 10.1016/S0042-6989(98)00285-5 10492833

